# High thermopower of mechanically stretched single-molecule junctions

**DOI:** 10.1038/srep11519

**Published:** 2015-06-26

**Authors:** Makusu Tsutsui, Takanori Morikawa, Yuhui He, Akihide Arima, Masateru Taniguchi

**Affiliations:** 1 The Institute of Scientific and Industrial Research, Osaka University, 8-1 Mihogaoka, Ibaraki, Osaka 567-0047, Japan

## Abstract

Metal-molecule-metal junction is a promising candidate for thermoelectric applications that utilizes quantum confinement effects in the chemically defined zero-dimensional atomic structure to achieve enhanced dimensionless figure of merit *ZT*. A key issue in this new class of thermoelectric nanomaterials is to clarify the sensitivity of thermoelectricity on the molecular junction configurations. Here we report simultaneous measurements of the thermoelectric voltage and conductance on Au-1,4-benzenedithiol (BDT)-Au junctions mechanically-stretched *in-situ* at sub-nanoscale. We obtained the average single-molecule conductance and thermopower of 0.01 *G*_0_ and 15 μV/K, respectively, suggesting charge transport through the highest occupied molecular orbital. Meanwhile, we found the single-molecule thermoelectric transport properties extremely-sensitive to the BDT bridge configurations, whereby manifesting the importance to design the electrode-molecule contact motifs for optimizing the thermoelectric performance of molecular junctions.

Electron transport in an organic molecule connected to metal leads has been extensively explored for applications in nanosensors and nanoelectronics^1^,^2^,^3^,^4^ In this virtually zero-dimensional nanostructure, simultaneous enhancement of electrical conductance and thermopower can be achieved by exploiting the steep peak in the density of states at the frontier molecular orbital levels, thereby opening new perspectives for achieving high thermoelectric figure of merit^5^,^6^ Owing to the advance in single-molecule technologies, it has become increasingly feasible to experimentally address the thermoelectric transport in individual molecular wires^7^,^8^,^9^,^10^ Despite the progress, however, while it is well-established that the single-molecule conductance varies widely depending on the molecular conformations and the electrode-molecule contact structures, little is known about the thermopower sensitivity on the molecular junction configurations, which is of fundamental importance for the development of efficient molecular thermoelectric devices^11^,^12^ In fact, geometry-sensitive nature of Seebeck coefficient was found in nanosystems similar to molecular junctions such as metal atom-sized contacts^13^,^14^.

Here we demonstrate statistical evaluation of geometric effects on the thermoelectric transport in 1,4-benzenedithiols (BDTs) anchored to Au nanoelectrodes at the single-molecule level. Au-BDT-Au bridge is known to possess a broad range of electric conductance due to the geometry-sensitive electronic coupling strength at the electrode-molecule links^15^,^16^,^17^, and therefore serves as a suitable model system for examining the structural dependence of thermoelectricity in a single-molecule wire.

## Results

### Simultaneous measurements of single-molecule conductance and thermoelectric voltage

**Figure 1:**
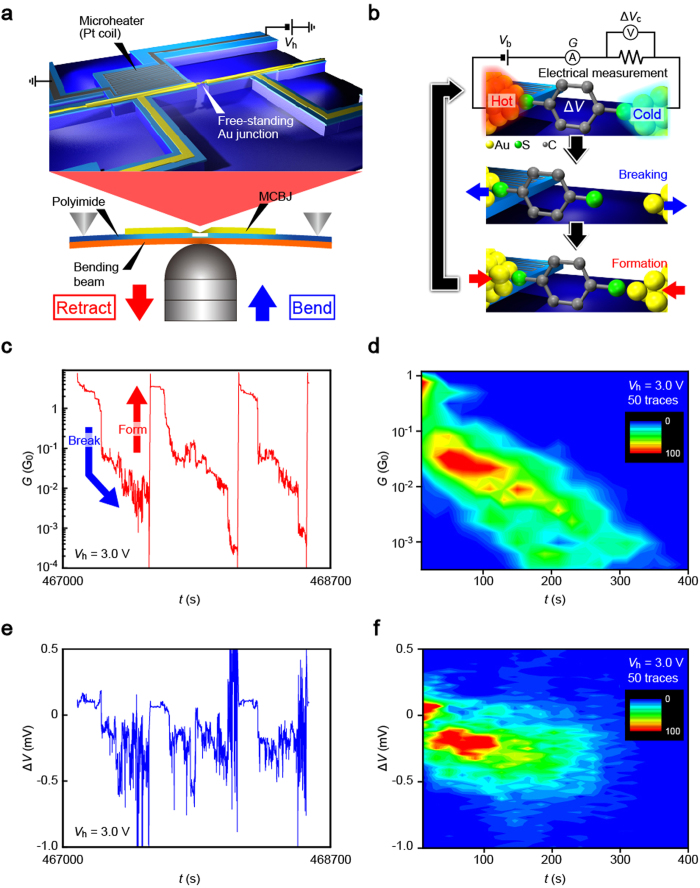
Simultaneous measurements of conductance and thermoelectric voltage of molecular junctions. (**a),** A microheater-embedded mechanically-controllable break junction used for forming stable Au-BDT-Au junctions at room temperatures. The free-standing Au junction was broken and reformed through manipulating the substrate bending via piezo-control. The adjacent Pt microheater was heated under the applied voltage *V*_h_ to create a temperature gradient at the junction for inducing detectable level of thermovoltage. (**b),** Schematic description of the measurement scheme. The conductance of molecular junctions was obtained by recording current under dc voltage *V*_b_ = 0.2 V. Subsequently, the potential drop at the 100 kΩ sensing resistor Δ*V*_c_ was acquired at *V*_b_ = 0 V, from which the thermovoltage at the junction Δ*V* was deduced. The sequential *G* - Δ*V*_c_ measurements were performed in the course of repeated formation and breaking of BDT molecular junctions. **c-d,** Partial *G* – *t* curve (**c**) and corresponding two-dimensional histogram (**d**) obtained at *V*_h_ = 3.0 V. **e-f,** The simultaneously recorded Δ*V* – *t* trace (**e**) and the two-dimensional histogram (**f)**.

A microheater-embedded mechanically-controllable break junction (MCBJ)^18^ was used to investigate the thermoelectric transport in Au-BDT-Au structures (Fig. 1a). In this device, a molecular-sized electrode gap can be formed by breaking Au nanobridge through mechanical deflection of the substrate. Then, BDT interconnects the nanoelectrodes whereby form a Au-BDT-Au junction. The ability to finely adjust the electrode positions enabled formation of many molecular bridges with different configurations in a short time allowing characterization of a variation in the thermoelectric transport in the molecular wires by utilizing the microheater to create a temperature gradient for inducing thermoelectric voltage there (Fig. 1b)^14^,^19^

The conductance *G* and thermoelectric voltage Δ*V* were simultaneously measured during stretching of BDT-decorated Au junctions (Figs S1-S2). We observed stepwise decrease in *G* as the Au nanocontact was narrowed by tensile deformation. Eventually, the junction was shrunk to one-atom size, which was confirmed by observations of long plateaus at 1 *G*_0_, where *G*_0_ = 2*e*^2^/*h* is the conductance quantum the electron mass *e* and Plank constant *h*. After breakage of the single-atom contacts, *G* dropped rapidly and showed another plateaus often with negative slope in a conductance range from 10^−1^ to 10^−4^ *G*_0_ (Fig. 1c). Conductance histograms revealed broad distributions but with several pronounced peaks at *n* × *G*_BDT_ (*n* = 1, 2, 3) with *G*_BDT_ = 0.011 *G*_0_ in accordance with the single-molecule conductance for Au-BDT-Au junctions reported in previous literatures^15^,^16^,^17^ (Fig. S5). The overall tendency can be also found in the two-dimensional histogram (Fig. 1d) where data are clustered at around 10^−2^ *G*_0_ corresponding to single- or a few BDT molecules bridging the Au nanoelectrodes^20^,^21^,^22^ On the other hand, the thermoelectric voltage showed fluctuations at around a certain value upon formations of the molecular junctions until *G* dropped to zero (Figs 1e-f and S6).

### Thermovoltage-conductance diagrams

**Figure 2:**
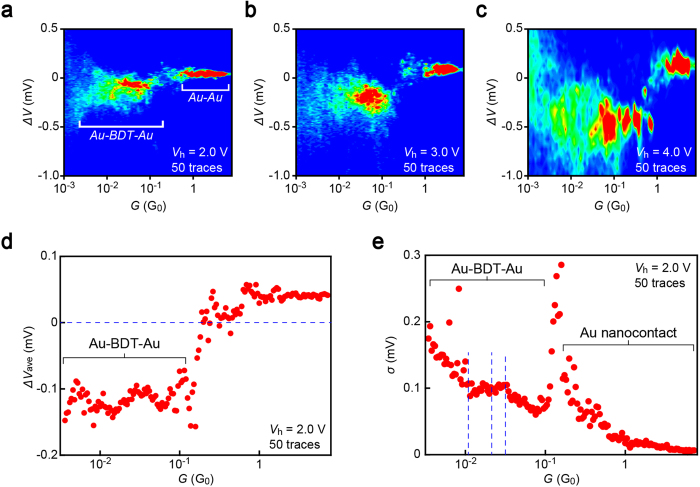
Single-molecule conductance versus thermoelectric voltage characteristics. **a-c,** Δ*V* – *G* diagrams of BDT junctions at *V*_h_ = 2.0 V (**a**), 3.0 V (**b**), and 4.0 V (**c**). **d,** Δ*V*_ave_ versus *G* semilog plots at *V*_h_ = 2.0 V. Dashed line denote Δ*V*_ave_ = 0 V. **e,** The standard deviation *σ* in Δ*V* plotted against *G*. Dashed lines represent *G* = *nG*_BDT_ for *n* = 1 to 3, where *G*_BDT_ = 0.011 *G*_0_ is the single-molecule conductance of Au-BDT-Au junctions. A sharp rise in *σ* at *G* ~ 0.2 *G*_0_ signifies a transition between Au atomic wires and BDT molecular junctions during the break junction experiments whereat the thermoelectric voltage changes its sign from positive to negative (see also Fig. S8).

Geometrical dependence of thermoelectricity in molecular junctions is investigated by constructing *G* – Δ*V* diagrams (Figs 2 and S7). The negative thermoelectric voltage of BDT molecular junctions tend to become higher with increasing heater voltage due to the larger temperature gradient created under elevated *V*_h_ (Fig. 2a–c). The average thermoelectric voltage Δ*V*_ave_ was found to change little over two orders of magnitude difference in *G* in the entire *V*_h_ range measured (Figs 2d and S8). This interesting feature would be interpreted by attributing the broad-range conductance to a formation of parallel circuits consisting of *n* BDTs with conductance *G*_BDT_, as the resulting thermoelectric voltage is essentially independent of the number of current-carrying molecules^7^. If this is the case, however, fluctuations in Δ*V* should become smaller at higher *G* since the measured thermovoltage would tend to be averaged by the number of molecules^10^, which is not consistent with that the standard deviation *σ* of Δ*V* changes little with *G* (Figs 2e and S10). The conductance-insensitive manner of the average thermoelectric voltage seems to be, therefore, an intrinsic property of BDT single-molecule wires.

### Average thermopower of BDT single-molecule junctions

**Figure 3:**
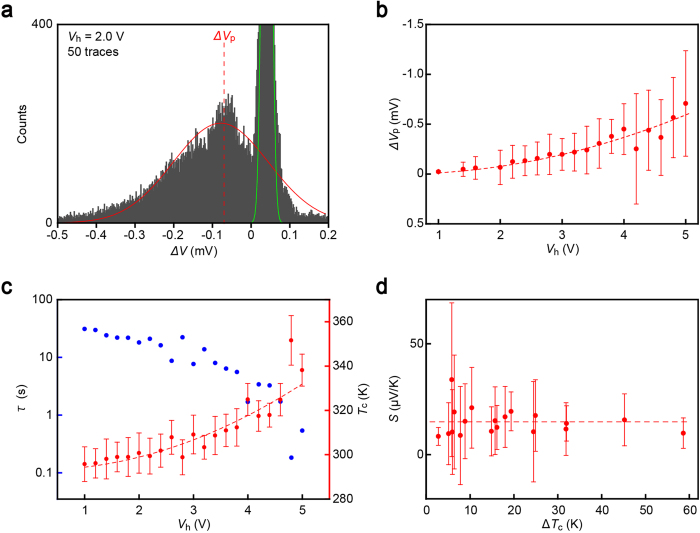
Single-molecule thermopower. **a,** Histogram of Δ*V* at *V*_h_ = 2.0 V. Peaks at the positive and negative regimes correspond to the thermovoltage occurred at Au atom-sized contacts and BDT molecular junctions, respectively. Red solid curve is Gaussian fitting to the thermoelectric voltage distribution that define the peak voltage Δ*V*_p_ for BDT single molecule junctions. Green curve is the fit to the thermovoltage distribution for Au contacts. **b,** Heater voltage dependence of Δ*V*_p_. The negative values reflect the thermoelectric characteristics of BDT junctions. Dashed lines are *V*_h_^2^ fits. Error bars denote the full-width at half-maximum of the *V*_p_ distributions. **c,** Au single-atom contact lifetime *τ* (blue) and the estimated local contact temperature *T*_c_ (red) plotted with respect to *V*_h_. Dashed line is a quadratic fit to *T*_c_. **d,** Thermopower *S* of BDT molecular junctions plotted against Δ*T*_c_. Dashed lines are the average of *S*.

To shed further light on the thermoelectric effects in Au-BDT-Au tunnelling junctions, we deduced the thermopower *S* from *V*_h_-dependence of the thermoelectric voltage. Δ*V* histograms show bimodal distribution (Fig. 3a). The broad feature at the negative region denote the expansive variation in Δ*V* of the BDT bridges. We extracted the peak values Δ*V*_p_ from each Δ*V* histogram, which demonstrate the typical thermoelectric characteristic of the molecular junctions, at various heater voltage conditions. Δ*V*_p_ – *V*_h_ correlation reveal steady increase in the average thermoelectric voltage with the heater voltage (Fig. 3b).

Derivation of single-molecule thermopower requires the temperature gradient *ΔT*_c_ at the junction. For this, we used the lifetime *τ* of Au atomic wires as an indirect thermometer to estimate the local temperature *T*_c_ at the vicinity of junctions^21^,^23^ *τ* was obtained from the plateau length within a conductance window of 0.8 *G*_0_ to 1.2 *G*_0_ in the *G* - *t* curves (Fig. S13), from which *V*_h_-dependence of *T*_c_ was assessed using Arrhenius expression *τ* = *f*_0_^−1^exp(*E*_B_/*k*_B_*T*_c_), where *f*_0_ = 3 × 10^12^ Hz, *E*_B_, and *k*_B_ denote the attempt frequency, the critical bond strength in the contact, and Boltzmann factor, respectively (Fig. 3c)^18^,^24^,^25^ Specifically, we performed linear fitting in the ln*τ* versus *V*_h_^2^ plots and deduced *E*_B_ from the fact that *T*_c_ = 293 K when *V*_h_ = 0 V. As a result, we obtained *E*_B_ = 0.82 eV. This value is slightly higher than the typical Au-Au bond energy in the single-atom chain^24^, which is attributable to enhancement of the contact stability via the dithiol molecules bridged in parallel^26^,^27^. Eventually, we acquired *ΔT*_c_ = *T*_c_ – *T*_0_ assuming that one side of the junction was kept at ambient temperatures *T*_0_ = 293 K (Fig. S3)^7^ and calculated the average thermopower of BDT single-molecule junctions through *S* = –Δ*V*_p_/Δ*T*_c_. We obtained positive thermopower of *S*_BDT_ = 15 ± 4 μV/K, the sign of which confirms dominant charge transport mechanisms in the two systems: hole tunnelling through the highest occupied molecular orbital (HOMO) level in the Au-BDT-Au system. Moreover, the present thermopower value is in good quantitative agreement with the previous experiment on many-molecule junctions^7^, which in turn indicates that the thermoelectric power of molecular junctions changes little with the number of constituent molecules. Note that *S*_BDT_ is a lower limit value as the temperature at the heat downstream of BDT junctions may be heated to above 293 K (Fig. S3).

### Single-molecule thermopower sensitivity on mechanical stretching

**Figure 4:**
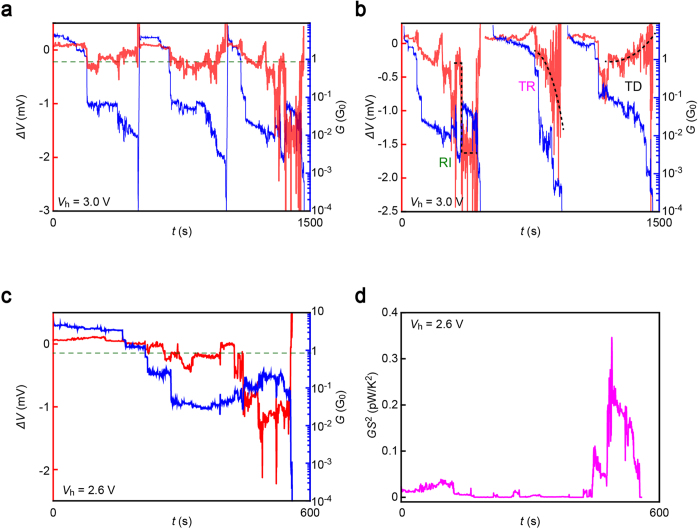
Mechanical response of thermovoltage. **a,** Three consecutive *G* (blue) and Δ*V* (red) traces at *V*_h_ = 3.0 V. Green dashed line indicates Δ*V*_ave_. Whereas the first two junctions possessed Δ*V* close to the average value, the third junction show a high thermovoltage exceeding Δ*V*_ave_ by more than an order of magnitude. **b,** Three types of stretching dependence of Δ*V* (red) in BDT junctions demonstrating rapid increase (RI), steady rise (TR), and steady decrease (TD) in the absolute value of the thermoelectric voltage by junction elongation. The graph also displays the simultaneously recorded *G* (blue). **c,** BDT junction with optimal thermoelectric properties observed in the present work that possess *S*_BDT_ = 120 μV/K and *G*_BDT_ = 0.21 *G*_0_ after RI. Red and blue curves denote the mechanical response of *G* and Δ*V*, respectively. **d,** Power factor *GS*^2^ calculated from data in (**c**).

The above results imply moderate impact of configurational degree of freedom on the thermoelectric effects in BDT junctions as a whole. However, it does not necessarily mean that there is no geometrical dependence; in sharp contrast, we often detected subtle change in the thermovoltage during mechanical stretching of Au-BDT-Au junctions in the respective traces (Fig. 4a). The electromechanical response could be classified into three groups: steady thermopower rise (TR) or decrease (TD) and rapid rise in *ΔV* entailing concomitant enhancement in *G* (RI) before the junction breakdown (Figs 4b and S13). It is noticeable that the conductance exhibits a steady decrease irrespective of the types of Δ*V* – *t* characteristics. This corroborates the predominant influence of molecule-electrode coupling on charge transmission probability in BDT junctions^15^,^16^,^28^, which on the other hand causes only minor effects on the thermoelectric power thereby giving rise to the *G*-independent behavior of Δ*V*_ave_^28^,^29^.

From practical viewpoints, the configurations achieved in RI events are a superior design of Au-BDT-Au junctions that realize two orders of magnitude improvement in the power factor *S*^2^*G* from the average value *S*_BDT_^2^*G*_BDT_ through concurrent enhancement of Δ*V* and *G*. In an optimal case (Figs. 4c and S13), we observed *S*_BDT_ = 120 μV/K and *G*_BDT_ = 0.21 *G*_0_. This particular BDT bridge attains a high power factor of 0.25 pW/K^2^ that is three orders of magnitude higher than 0.19 fW/K^2^ calculated from *S*_BDT_ = 15 μV/K and *G*_BDT_ = 0.011 *G*_0_ of the average junctions. The present work thus suggests the importance of engineering not only the molecular structure but also the atomic junction configurations for pursuing high-performance single-molecule thermoelectrics.

## Discussion

It is intriguing that the average thermopower of BDT single-molecule junctions changes little over the broad range of the conductance. In bulk materials, the thermopower is described by the Mott’s theory that states *S* ~ 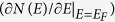/*N*(*E*_F_), where *N*(*E*) and *E*_F_ is the density of states at the energy *E* and the Fermi level, respectively^30^. This generally leads to a tradeoff between *G* and *S* as an improvement in the conductivity by increased *N*(*E*_F_) will be compensated by accompanying diminishment in the thermopower. The tunability of the single-molecule thermopower independently from the conductance may thus enable a unique way of engineering the thermoelectric properties of molecular junctions.

**Figure 5:**
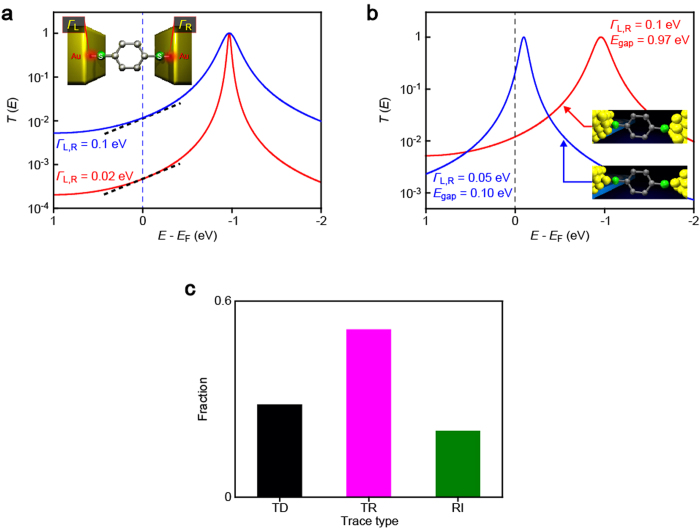
Geometrical dependence of thermoelectric voltage in single-molecule junctions. **a,** Coupling-insensitive thermopower of BDT tunnelling junctions. A transmission curve calculated for the average properties of *S*_BDT_ = 15 μV/K and *G*_BDT_ = 0.011 *G*_0_ (blue) using Lorentzian expression of the transmission 
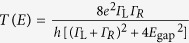
 and the thermopower 
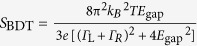
 with *E*_F_ = 5.0 eV, where *E*_gap_ is the HOMO-Fermi level gap and *Γ*_L,R_ is the coupling strength at the left and right electrodes. While the curve tends to become sharper under lower *Γ* (red), the slope of the transmission curve at the electrode Fermi level *E*_F_ (black dotted lines) changes little due to the relatively large *E*_gap_ of *E*_gap_ = 0.97 eV. **b,** Transmission curve for BDT junctions having *E*_gap_ = 0.10 eV with *Γ*_L,R_ = 0.05 eV (blue) as deduced from *S*_BDT_ = 120 μV/K with *G*_BDT_ = 0.21 *G*_0_ attained by a specific junction displayed in Fig. 4c. A curve for the average junctions is also shown (red). The schematic models denote possible junction motifs responsible for the two different thermoelectric properties having a hollow-hollow geometry and an adatom-coordinated configuration. **c,** The normalized number of events for the three Δ*V – t* characteristics.

The *S* - *G* characteristics is interpreted by considering that HOMO of Au-BDT-Au junctions lies ~ 1 eV away from the electrode _F_ermi level *E*_F_^28^. The thermovoltage in the molecular tunnelling junctions is determined by the slope of transmission curves near *E*_F_, *i.e.* Δ*V* ~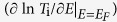, where *T*_i_ is the charge transmission^29^. On the other hand, the extensive range of *G* is anticipated to be stemming from a variation in the coupling strength at the left *Γ*_L_ and right *Γ*_R_ sides of the molecule-electrode contacts engendered by a stochastic nature in the contact mechanics^15^,^16^. While stronger coupling tends to broaden the transmission curve, the contribution on *S* is less significant for a metal-molecule-metal junction with a large *E*_F_-HOMO energy gap *E*_gap_ (Fig. 5a)^28^,^29^. Hence, the constant average thermopower suggests reproducible formations of BDT single-molecule junctions with large *E*_gap_ but different degrees of *Γ*_L,R_.

More quantitatively, when estimating *E*_gap_ and *Γ*_L,R_ (BDTs are assumed to couple equally to both sides of Au electrodes) from the simplified Landauer expression of *S*_BDT_ and *G*_BDT_ in the weak coupling regime (i.e.*, E*_gap_ < < *Γ*_L,R_),^31^ we obtain *E*_gap_ = 0.97 eV and *Γ*_L,R_ = 0.10 eV for the typical BDT junctions with *S*_BDT_ ∼ 15 μV/K (Fig. 5a,b), which is in good agreement with the previous works^28^,^29^ Compared to the average BDT bridges, the high-*S* single-molecule junction (Fig. 4d) possesses much smaller HOMO-*E*_F_ energy gap of 0.10 eV with weaker electronic coupling strength, *Γ*_L,R_ = 0.05 eV (Fig. 5b).

We note that recent experiments by Kim *et al.*^16^ report narrower *E*_gap_ of about 0.3 eV, which is in good agreement with GW calculations^32^. Although this discrepancy may stem from the simple model we used, i.e. single-particle model with symmetric coupling, it is difficult to directly compare the energy gap we obtained with the value in the literature as the approaches for deriving the energy gap are quite different in the two works (thermopower or *I*-*V*) together with the temperature conditions used (above 300 K or 4.2 K) that should give rise to different contact mechanics and hence different junction geometries are quite different. Further effort should be thus devoted to address the quantitative accuracy of the analysis method described here.

The geometrical dependence of thermoelectricity in Au-BDT-Au junctions is inferred in the Δ*V – t* profiles. TR and TD tendencies clearly suggest accompanying roles of the mechanical stretching along specific directions, which results in molecular tilting toward an upright conformation and elongation of the Au-S bond lengths of BDT in a Au-hollow or -top geometry, to weaken the electrode-molecule electronic coupling that leads to steady decrease in *G* and meanwhile inducing gradual shift of HOMO toward or away from *E*_F_, respectively^33^,^34^,^35^,^36^. On the other hand, the rapid increase in both *G* and Δ*V* of RI indicates a drastic change in the Fermi alignment: *E*_gap_ lowered for more than 0.5 eV and *Γ*_L,R_ decreased by up to 0.05 eV. This anomalous feature presumably stems from over-elongated Au-S bonds of mechanically-stretched BDT junctions and accompanied electrode-to-thiol charge transfer that would provide additional MOs near *E*_F_ with weak electronic coupling, and hence small *E*_gap_ and *Γ*_L,R_. It was in fact demonstrated theoretically that thermopower increases rapidly under stretching just before junction breakdown^36^. Furthermore, the fact that BDT junctions tended to dissociate in a relatively short period of time after the RI events infers the contact mechanics involved therein: unstable electrode-molecule link is formed at the Au-S contacts probably having an adatom-coordinated metastable motif with relatively long Au-S distance through pulling-out of the Au surface atoms during mechanical stretching^17^,^36^ For this to happen, molecular junctions are required to endure the forces and thermal fluctuations until the contact is departed at a certain extent, which is likely to be a rare case as Au-BDT-Au structure fractures spontaneously at the Au-Au contacts in vicinity of the Au-S link as illuminated by that the RI feature was found in only about 20 % of the traces measured (Fig. 5c). We expect that future works will shed more light on the physics underlies this intriguing effect found in the stretched molecular junctions.

## Methods

### Fabrication of heater-embedded MCBJs

A phosphor-bronze beam of 0.5 mm thickness was covered with a 4 μm thick polyimide layer. We then formed Au micro-leads on the polyimide using photolithography and radio-frequency magnetron sputtering processes followed by lift-off in *N,N*-dimethylformamide. After that, Al_2_O_3_ heat sinks were fabricated through delineating two island patterns by an electron-beam lithography with ZEP-520A-7 resist, subsequent inductively-coupled plasma sputtering for depositing a 40 nm thick Al_2_O_3_ layer, and the lift-off procedure. A Pt coil and a Au nano-junction were further prepared on the Al_2_O_3_ regions by the electron-beam processes and the sputtering. After the nanofabrications, the sample substrates were exposed to isotropic reactive ion etching with oxygen etchant gas for sculpting the polyimide. As a result, we obtained a free-standing Au junction of length approximately 2 μm. On the substrate, a Teflon ring was fixed on the substrate surface using polyimide as glue, which was used to fill in a molecular solution in prior to the break junction experiments. The cross-plane heat leakage was effectively suppressed by the thick polyimide layer that has excellent thermal insulating properties. Meanwhile, the Al_2_O_3_ layers served as effective thermal link to conduct the Joule heat at the Pt coil to the Au junction for generating finite temperature gradient to induce measureable thermoelectric voltage^16^.

### Formations of BDT single-molecule junctions

A Teflon ring cell on a MCBJ substrate was filled with a dilute toluene solution of BDT molecules adjusted to a concentration of 1 μM. Toluene and BDT were purchased from Aldrich Co. and utilized as-received. The MCBJ bending beam was then bent by a three-point bending mechanism to adhere BDT molecules on the fresh breakage Au surface. The chamber was then evacuated to remove the solvent while leaving BDTs strongly bound to the Au surface via Au-S bonds. When the vacuum reached below 10^−5^ Torr, we started the single-molecule measurements at room temperatures under various *V*_h_ conditions. In this, the BDT-decollated Au junction was broken and formed repeatedly in a controlled manner by manipulating the motion of a piezo-driven pushing rod (Fig. S1). At the same time, the temporal change in the junction conductance and the potential drop at the sensing resistor were monitored.

### Thermoelectric voltage and electrical conductance measurements

The potential voltage drop at the sensing resistor Δ*V*_c_ and the junction conductance *G* were simultaneously recorded under constant *V*_h_ conditions when the junction conductance was below 8 *G*_0_ in course of the break junction experiments. A picoammter/source unit (Keithley 6487) was used to obtain *G* under a dc voltage *V*_b_ = 0.1 V. After collecting *G, V*_b_ was set to 0 V and Δ*V*_c_ was measured by a nanovoltometer (Keithley 2182A). The measurement system and the motion of the piezo-element beading the MCBJ substrate were GPIB-controlled for automated measurements of many BDT single-molecule junctions under various *V*_h_ conditions (Fig. S2). Because of the small thermal current in the high-resistance molecular tunnelling junctions, which is on the order of pA, the integration time necessary for the nanovoltometer to acquire the thermoelectric voltage with accuracy exceeds 0.1 seconds. The overall measurement time to record *G* and Δ*V*_c_ was therefore as long as 0.3 seconds. Under this circumstance, a slow junction stretching rate of 6 pm/s was used to induce the mechanical deformations of Au-BDT-Au junctions, as it is critically important to preserve the junction status, including the molecule-electrode configurations and the molecular conformations, for prolong time to enable measurements of the thermopower and the electrical conductance for each molecular junctions with distinct geometries.

### Data analysis

The junction conductance *G* was obtained by subtracting the 100 kΩ series resistance from the measured conductance. The actual thermoelectric voltage Δ*V* occurring at the junction was deduced from Δ*V* = Δ*V*_c_(1 + 10^−5^/*G*) considering voltage division at the sensing resistor after background subtraction (Fig. S2). The background Δ*V* was calibrated at every measurement for each *V*_h_ condition by collecting *G* and Δ*V*_c_ at *V*_h_ = 0 V for 10 trials of the repeated junction formation/breaking processes in the beginning.

## Additional Information

**How to cite this article**: Tsutsui, M. *et al.* High thermopower of mechanically stretched single-molecule junctions. *Sci. Rep.* **5**, 11519; doi: 10.1038/srep11519 (2015).

## Supplementary Material

Supplementary Information
