# Supplementary material for: High thermopower of mechanically stretched single-molecule junctions
Source: Sci Rep. 2015 Jun 26;5:11519. doi: 10.1038/srep11519 (PMC4481826; doi:10.1038/srep11519)
Supplement: Supplementary Information [file srep11519-s1.pdf]

Supplementary Information for

High thermopower of mechanically stretched single-molecule junctions

Makusu Tsutsui, Takanori Morikawa, Yuhui He, Akihide Arima & Masateru Taniguchi

The Supplementary Information includes:

1. Repeated formations of Au-BDT-Au molecular junctions (Fig. S1)

2. Simultaneous measurements of thermoelectric voltage and conductance (Fig. S2)

3. Temperature distributions in a microheater-embedded MCBJ (Figs. S3-S4)

4. Supplementary Figures (Fig. S5-S13)

5. Supplementary references

1. Repeated formations of Au-BDT-Au molecular junctions

Au-1,4-benzenedithiol (BDT)-Au junctions were formed by using a microheater-embedded mechanically-controllable break junction (MCBJ). In this device, a free-standing Au nanocontact was mechanically broken through the substrate deflection by a three-point bending mechanism (Fig. S1a). Here, we used a piezo-actuator to push the MCBJ beam from the back side that moves in a vertical direction by the applied dc voltage V_{piezo} at a rate 1 μm/V. Furthermore, the device configuration was designed to provide the attenuation factor $r = \eta d_j / D_{piezo} = 3 \times 10^{-4}$, where d_j is the tensile displacement of the Au junction[S1,S2] induced by the beam bending through the motion of the piezo-actuator by a distance D_{piezo} and η is a coefficient describing the mechanical deformation of the polyimide layer.[S3] This enables fine control of the contact mechanics at a sub-picometer resolution thereby allowing reproducible formations of stable atomic and molecular junctions.[S4]

In prior to the single-molecule measurements, the MCBJs were immersed in a dilute toluene solution of BDT (1 μM). The junction was then broken at the narrowest constriction through the substrate bending to let BDT molecules adsorb on the fresh Au

breakage surface exposed to the solution via Au-S links. The chamber was then evacuated to remove the solvent for preventing molecular aggregation on the junction.[S5]

In experiments, the Pt microheater adjacent to the Au nanobridge (Fig. S1b) was heated by applying a dc voltage V_h and the MCBJ substrate was bent at varying speeds at above 1 nm/s depending on the conductance states G during junction elongation until the Au contact was narrowed mechanically to a size of a few atoms with G below 6 G_0. Thereafter, on the other hand, the junction stretching speed was set to 6 pm/s. Under this slow stretching speed condition, the Au atomic contacts underwent thermoactivated spontaneous breakdown showing long 1 G_0 plateaus in G - t traces that last for longer than 10 seconds.[S6] After the junction fracture, BDT molecules were often bridged the thus formed two Au nanoprobes, which was observed as another plateaus at a low-G regime in a range from 0.1 G_0 to 0.001 G_0 (Fig. S1c). Meanwhile, we recorded the thermoelectric voltage and the conductance of the junctions when G decreased below 8 G_0 (Fig. S1d). Further stretching, G dropped to below 10^{-4} G_0 indicating BDT molecular junction breakdown at the Au-S bonds. Subsequently, the junction was closed at a rate 1 nm/s until G exceeded 15 G_0. The entire processes were repeated for 50 times at each

V_h condition from 1.0 V to 5.0 V. All the measurements were conducted at room temperatures in a vacuum better than 10^{-5} Torr.

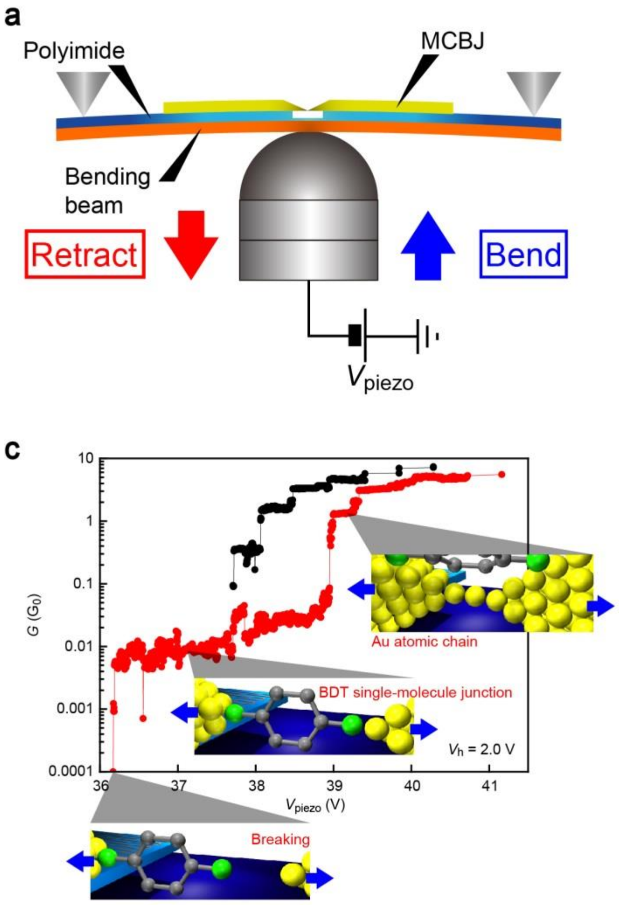

Figure S1. Repeated formation of Au-BDT-Au junctions. a, A schematic illustration of a MCBJ system. A piezo-driven pushing rod was used to bend the substrate that moves vertically under the voltage V_{piezo} at a rate 1 μm/V. **b,** Scanning electron micrograph of a microheater-embedded MCBJ consisting of a free-standing Au junction and a Pt microheater on Al_2O_3 layers patterned on a

polyimide-coated phosphor bronze substrate. **c-d,** The conductance *G* (c) and the thermoelectric voltage Δ*V* (d) obtained during the repeated break junction experiments. Red and black plots denote the two consecutive traces recorded. Only a part of the data is shown for the second trace (black) for the sake of clarity.

2. Simultaneous measurements of thermoelectric voltage and conductance

The thermoelectric voltage at the atomic and molecular wires $\varDelta V$ were acquired by measuring the potential drop $\varDelta V_c$ at the 100 kΩ sensing resistor connected in series to the junction together with the conductance G (Fig. S2a).[S7] Briefly, we first recorded G under the dc voltage $V_b = 0.2$ V applied to the junction when $G < 8\ G_0$ in course of the aforementioned junction stretching processes. Subsequently, we switched off the voltage source and measured ΔV_c. The sequential recording was performed until G decreased from 8 G_0 to 0.0001 G_0. A constant bias voltage was imposed to the microheater throughout the measurement to create a temperature gradient at the junction for inducing measurable amount of ΔV_c. Because of the long integration time required for measuring the small thermoelectric voltage at the high-resistance molecular junction with accuracy, the sampling rate of G and ΔV_c was as slow as 3 Hz.

The measured thermoelectric voltage was found to include a background presumably stemming from the thermoelectric effects at the bulk interconnects.[S7] This background voltage was calibrated by acquiring ΔV_c at $V_h = 0$ V for 10 junction formation/breaking processes at every each V_h condition measured (red plots in Fig. S2b for example). By subtracting the thus obtained background from ΔV_c, we deduced the actual thermoelectric

voltage occurring at the junction by taking into account the voltage division in the circuit as $\Delta V = \Delta V_c(1 + 10^{-5}/G)$ (Fig. S2c).[S7]

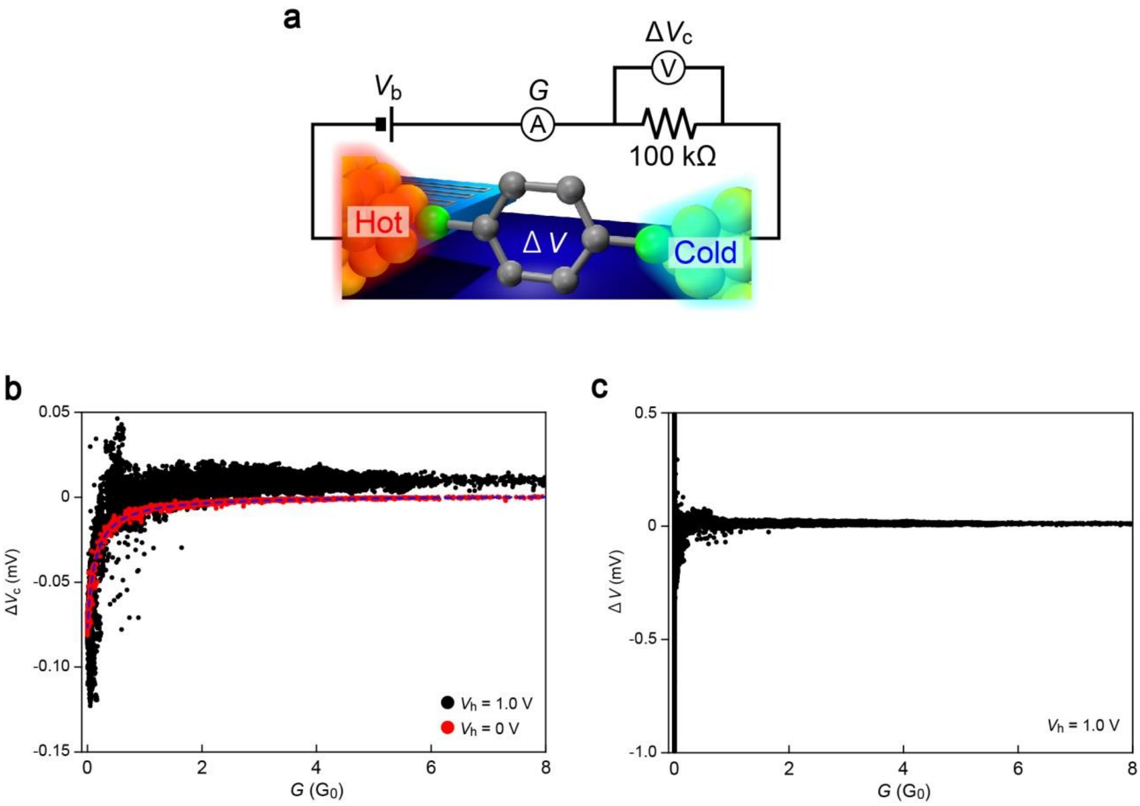

Figure S2. Measurements of conductance and thermoelectric voltage of Au atomic wires and Au-BDT-Au molecular junctions. a, An electrical circuit used for the simultaneous measurements of the conductance G and the thermoelectric voltage ΔV. It is noticed that the measured ΔV_c is a potential drop at the 100 kΩ sensing resistor connected in series to the junction. We calculated ΔV at the contact considering the voltage division as $\Delta V = \Delta V_c(1 + 10^{-5}/G)$. **b,** ΔV_c versus G scatter plots at $V_h = 1.0$ V. Red plots

are the data for calibration corrected at V_h = 0 V. **c,** ΔV at V_h = 1.0 V obtained from ΔV_c by subtracting the background at V_h = 0 V.

3. Temperature distributions in a microheater-embedded MCBJ

Heat transfer in the microheater-embedded MCBJs were simulated theoretically by COMSOL. The three-dimensional structure was defined following the actual dimension and material used: A polyimide layer of thickness 4 μm was put on a phosphor bronze substrate; on the polyimide, 40 nm thick Al_2O_3 layers were drawn; and on the top lies a Au junction of thickness 100 nm with a platinum microheater adjacent to its left side at a distance of 300 nm. The acute angles at the tips of the Au junction were set to 45° and the narrowest constriction was defined to be 50 nm to 1 nm wide. In the simulation, we set the temperature of the microheater at T_h = 500 K while assuming the bottom plane of the 5 μm phosphor-bronze layer at room temperature T_c = 293 K. A heat transfer in solids module of COMSOL has been employed to estimate the temperature distribution, and the simulation results are displayed in Fig. S3.

The results show a substantial temperature drop at the junction due to the geometrical constriction. It is also noticeable in Fig. S3c that heat leakage through the substrate is sufficiently suppressed in a direction along the junction due to the free-standing structure together with the micrometer-scale deep etching of the polyimide. Figure S3e illustrates the temperature distribution along the axis pointing from higher temperature to the lower

one for junctions having a constriction of size 50 nm, 10 nm, or 1 nm (We note that the present simulation is not valid for 1 nm contacts as it calls for a theory that fully takes quantum effects into account; we show the result here just to show the qualitative tendency). It gives an average temperature gradient of 3.2 K/nm at the junction having a contact of size 50 nm × 50 nm. Although the actual temperature profile cannot be obtained for molecular junctions, these results ensure that substantial amount of temperature difference would be established at the atomic and molecular bridges considering their low thermal conductance compared to the large Au contacts.

While the thermal analysis reveals the capability of the microheater to create a Kelvins of temperature difference at the junction, Fig. S3 also indicates that the temperature drops largely in the micro-scale Au lead. This makes the thermovoltage generated in the Au lead to be canceled there as described in the previous work.[S7]

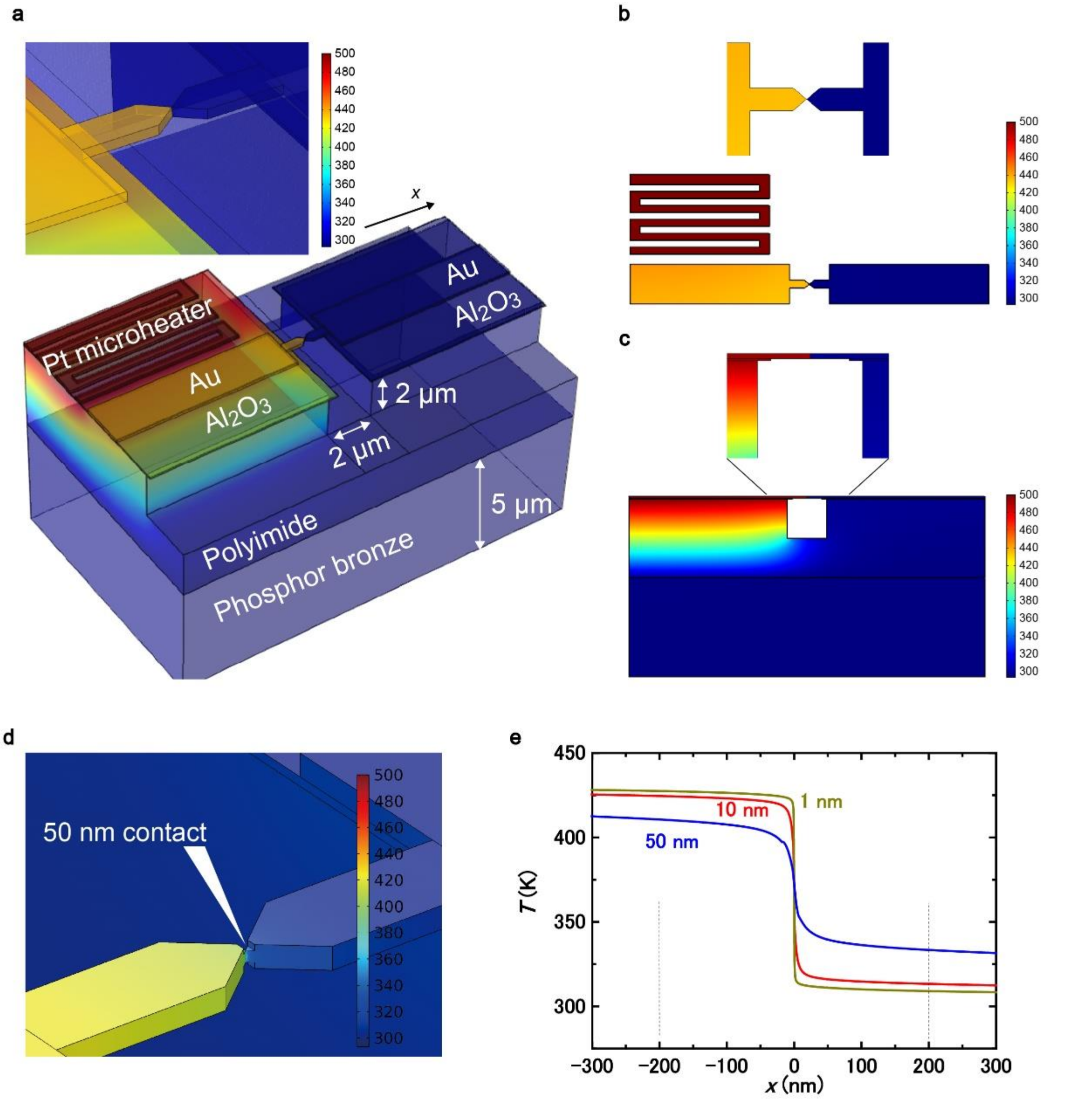

Figure S3. Heat transfer analysis for a microheater-embedded MCBJ. a, A three-dimensional model of a microheater-embedded MCBJ having a 1 nm-sized Au contact and the temperature distributions around the junction in case when the temperature T_h at a Pt microheater was set to 500 K. **b-c,** Top (b) and side views (c) of the temperature distributions at T_h = 500 K. **d,** Three-dimensional model showing a magnified view of a Au junction in a microheater-

embedded MCBJ. The contact size is narrowed to 50 nm scale in the image. **e,** Temperature profile along the junction with a contact of size 50 nm x 50 nm (blue), 10 nm x 10 nm (red), and 1 nm x 1 nm (yellow) modeled at the middle. Note that the temperature at the heat downstream tends to be closer to 293 K as the contact size become smaller. The inset shows a magnified image of the junction with dotted lines denoting the 500 nm-long constriction. The position of the middle part of the junction was taken to be x = 0.

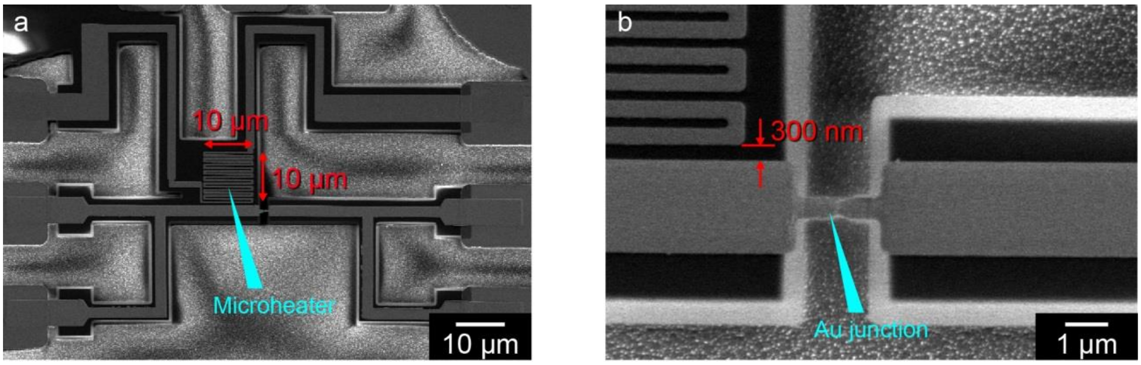

Figure S4. Dimensions of a microheater-embedded MCBJ. a-b, Scanning electron micrographs of a microheater-embedded MCBJ. A Pt coil is fabricated in a 10 μm x 10 μm region at one side of the Au lead. The distance between the Au lead and the Pt coil is made to be about 300 nm.

4. Supplementary figures

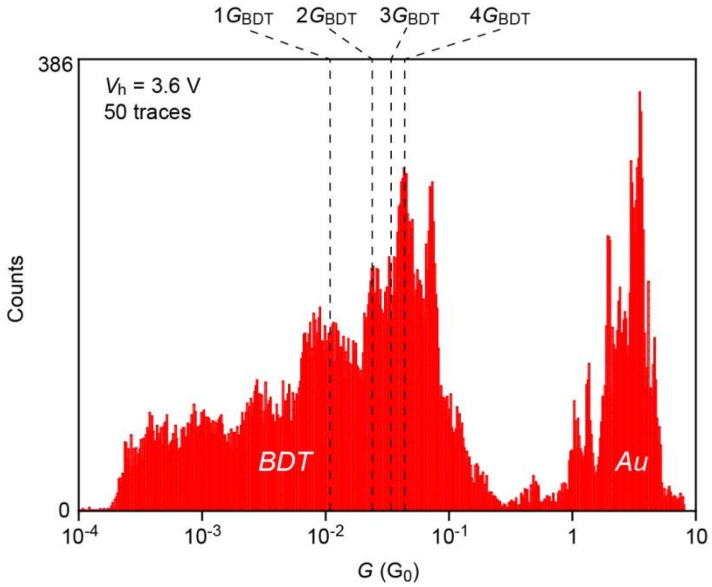

Figure S5. A typical conductance distribution of BDT-Au-BDT molecular junctions. The peaks at G greater than 1 G_0 represent the conductance states of Au atomic wires whereas those below 1 G_0 are attributed to the conductance of BDT molecular junctions. Dotted lines show conductance states at the multiple integers of single-molecule conductance G_{BDT} = 0.011 G_0 reported in the previous literatures.[S4,S8,S9]

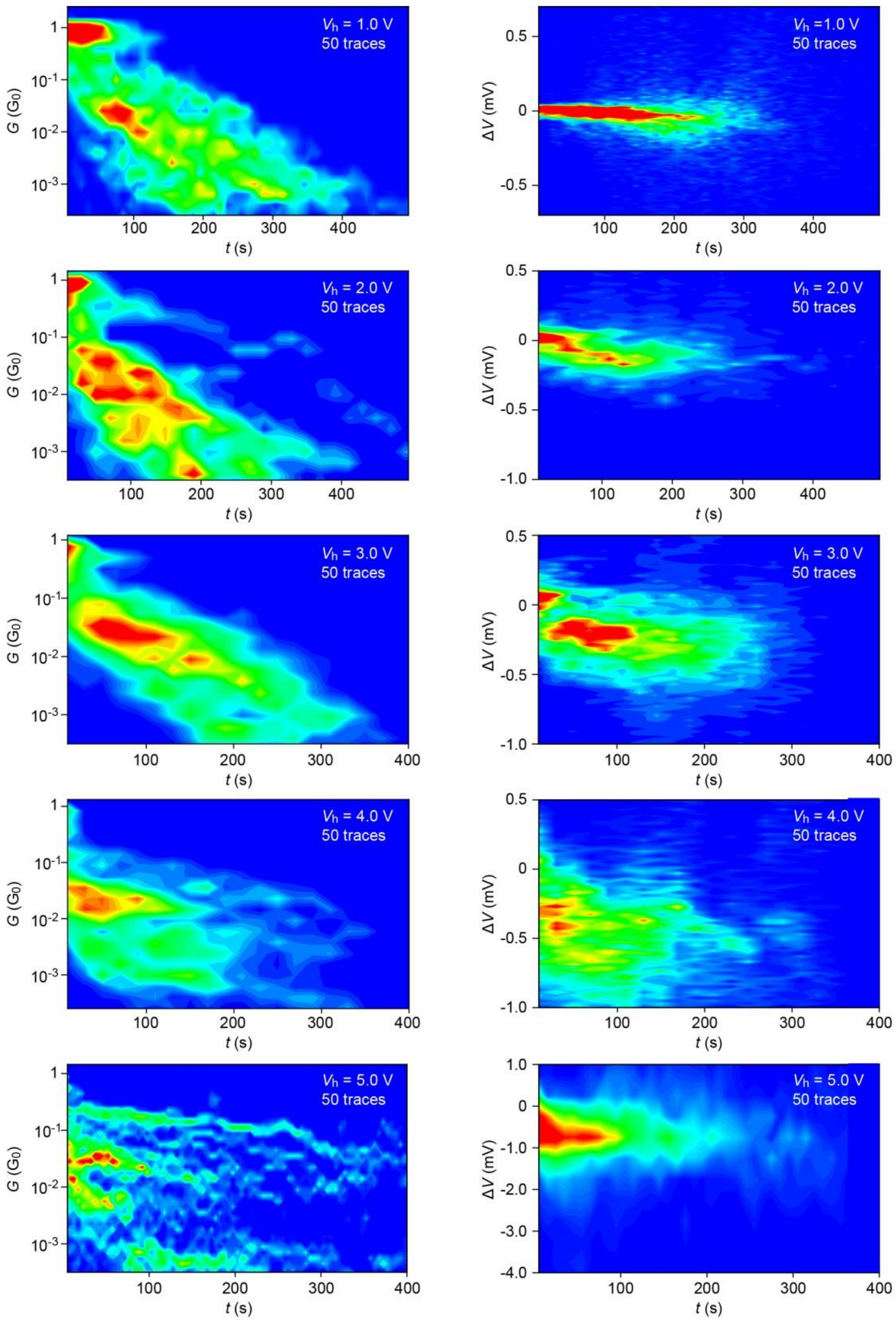

Figure S6. Time traces of conductance and thermoelectric voltage. Two-dimensional histograms of the conductance G and the thermoelectric voltage ΔV

as a function of time for Au-BDT-Au junctions under stretching at V_h from 1 to 5 V.

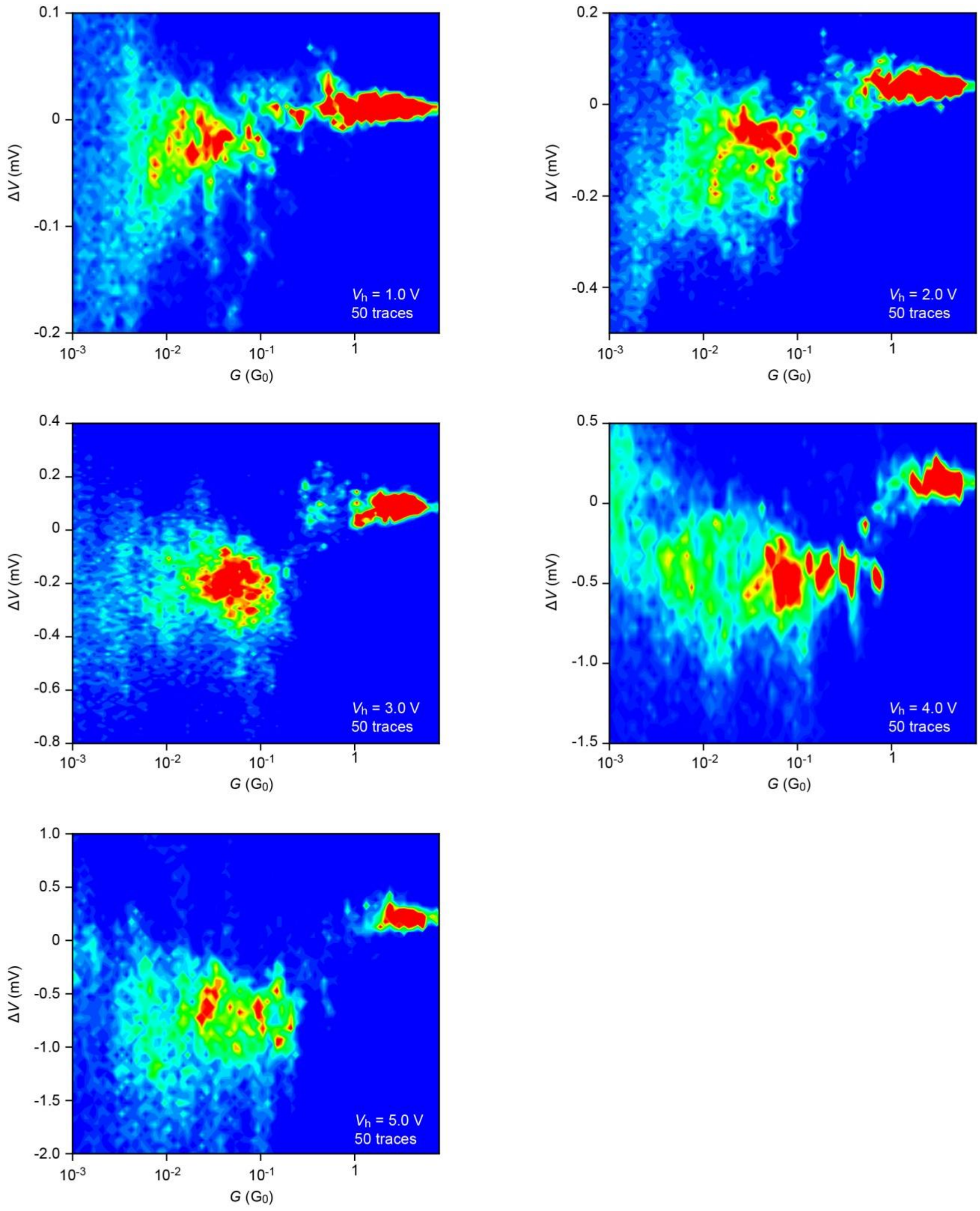

Figure S7. Conductance versus thermoelectric voltage two-dimensional histograms at various V_h conditions. Positive and negative ΔV is found at G

> 1 G_0 and G < 0.1 G_0, which are ascribed to thermoelectricity in Au atomic wire and BDT molecular junctions, respectively. The thermovoltage is rising steadily with increasing V_h suggesting larger temperature gradient under higher V_h.

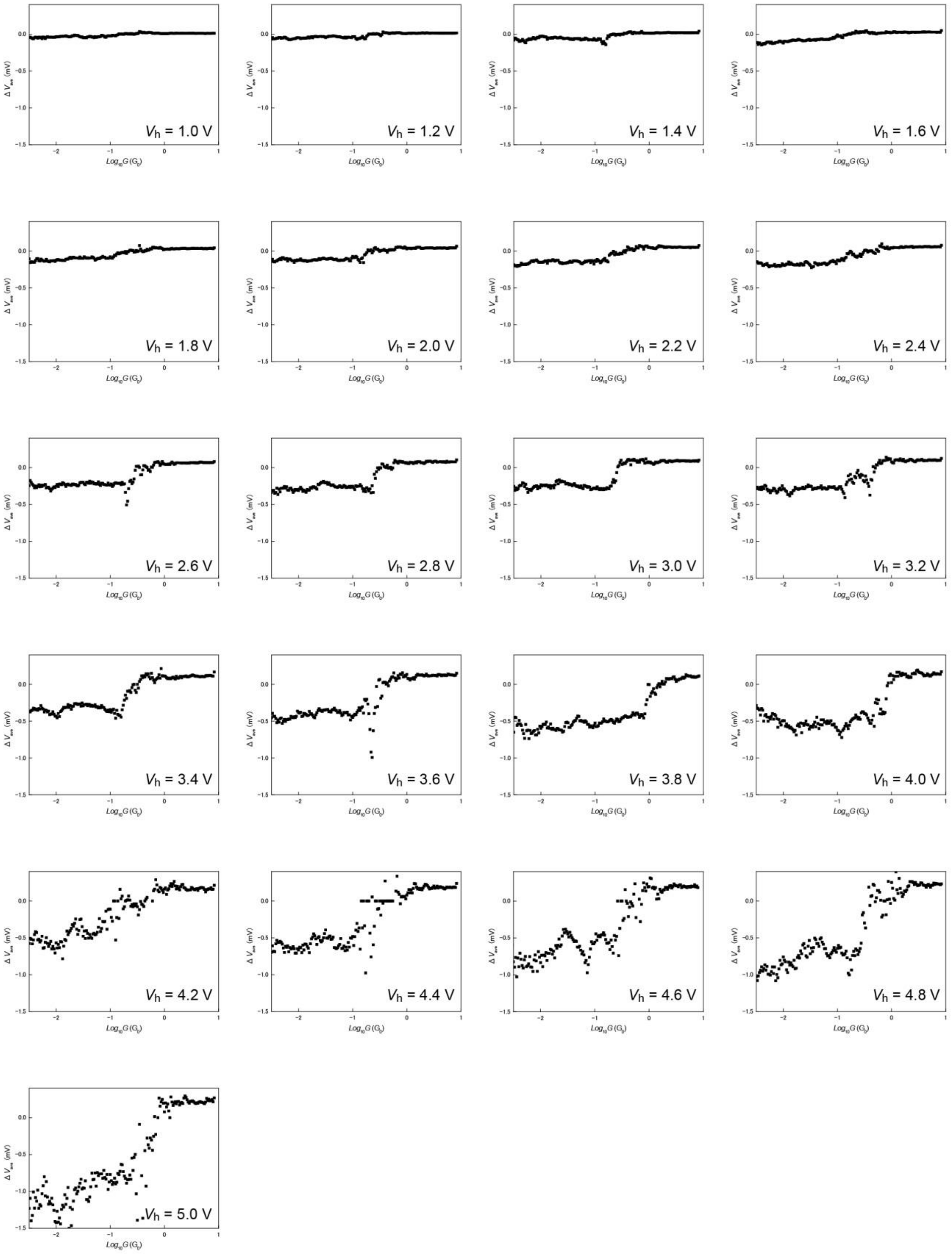

Figure S8. Average thermoelectric voltage. The average thermoelectric voltage ΔV_{ave} plotted against $Log_{10}G$.

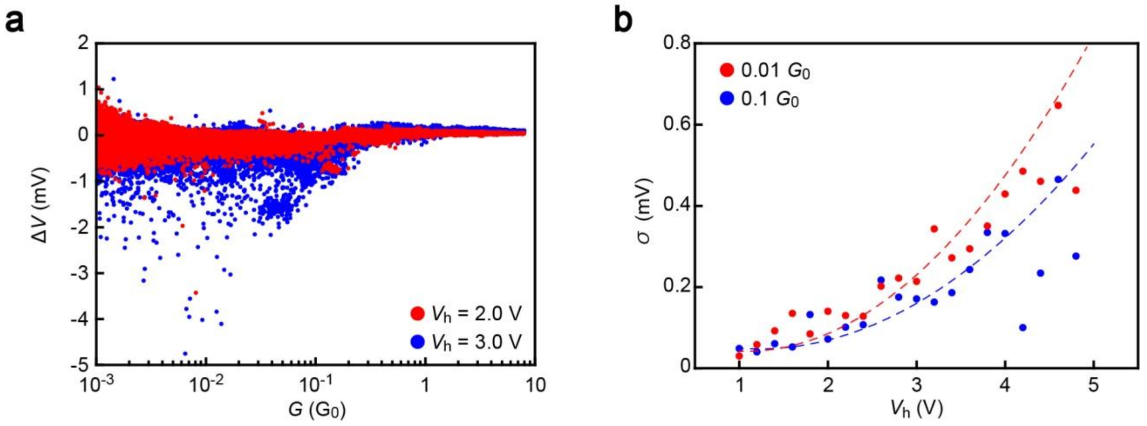

Figure S9. Fluctuations in the thermoelectric voltage. a, Scattering plots of ΔV with respect to G for data obtained at V_h = 2.0 V (red) and 3.0 V (blue). **b,** A heater voltage dependence of the standard deviation σ of ΔV within a conductance window of 0.02 G_0. Red and blue plots are σ at G = 0.01 G_0 and 0.1 G_0, respectively. Steady increase in σ with V_h indicates enlarged thermovoltage fluctuations under elevated temperature that facilitates junction structural changes and give larger thermal noise.

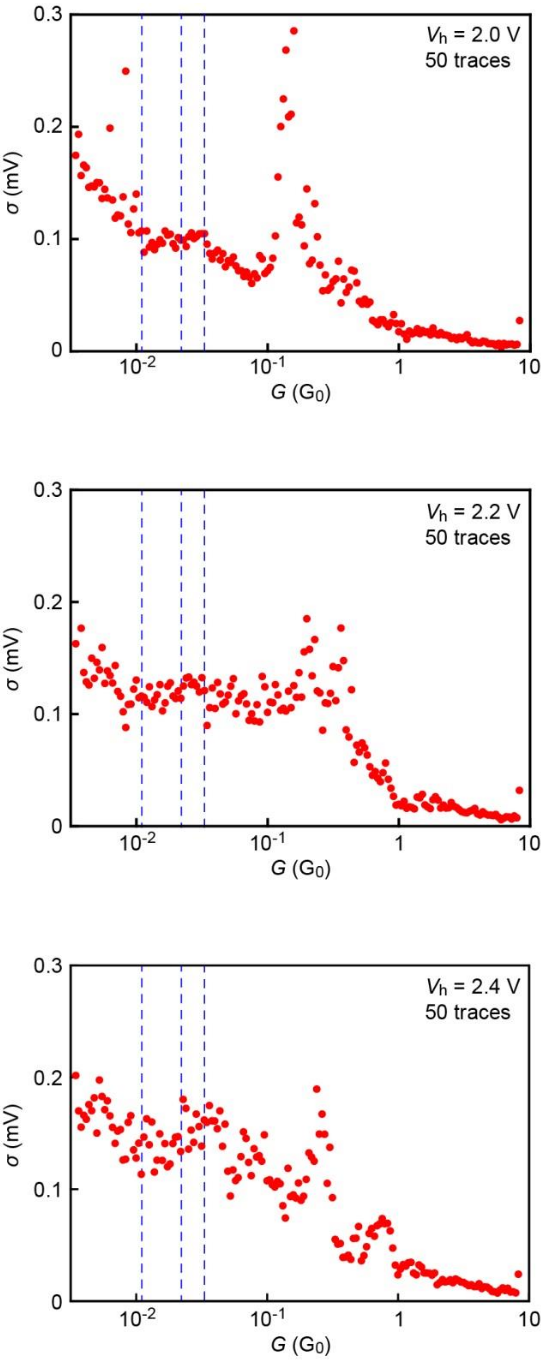

Figure S10. Conductance dependence of the thermoelectric voltage fluctuations. Dotted lines denote $n\ G_{BDT}$ (n = 1, 2, 3) where G_{BDT} = 0.011 G_0 is the representative single-molecule conductance of Au-BDT-Au junctions.[S4,S8,S9]

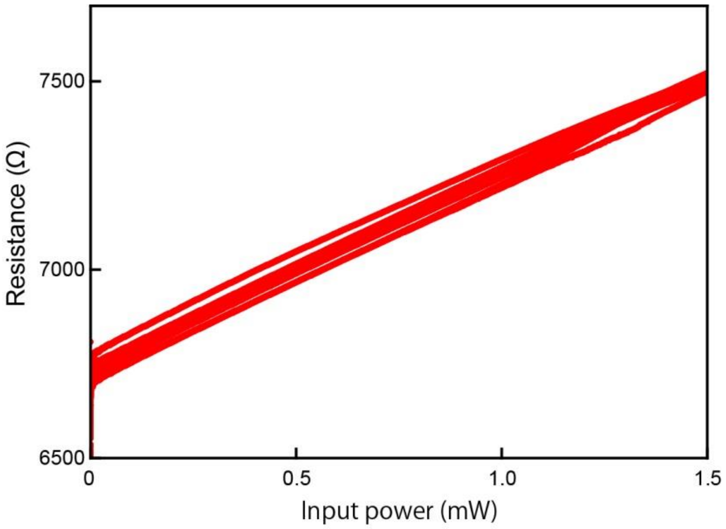

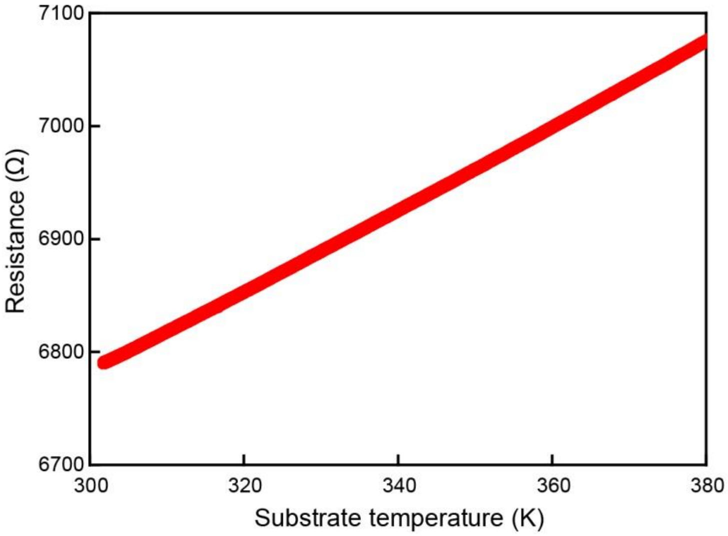

Figure S11. Calibration of the Pt microheaters. Top, A linear increase in the resistance of the microheaters with the input power caused by Joule self-heating. **Bottom,** Linear dependence of the microheater resistance to the substrate temperature. These results indicate linear (quadratic) rise in the local temperature at the microheaters with the input power (heater voltage).[S7]

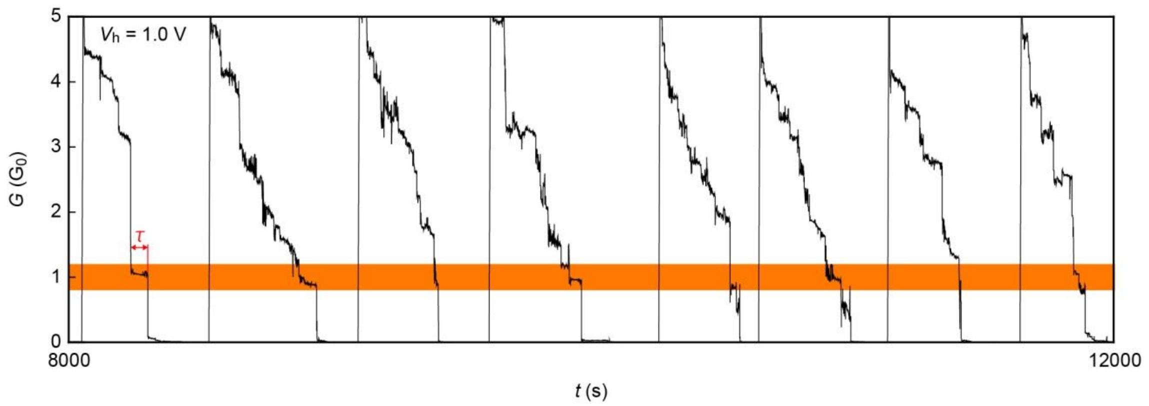

Figure S12. Estimation of the Au single atom contact lifetime. The single-atom contact life time *τ* was obtained by measuring the persistence time of *G* to be kept in a window from 0.8 G_0 to 1.2 G_0 indicated by the orange band.

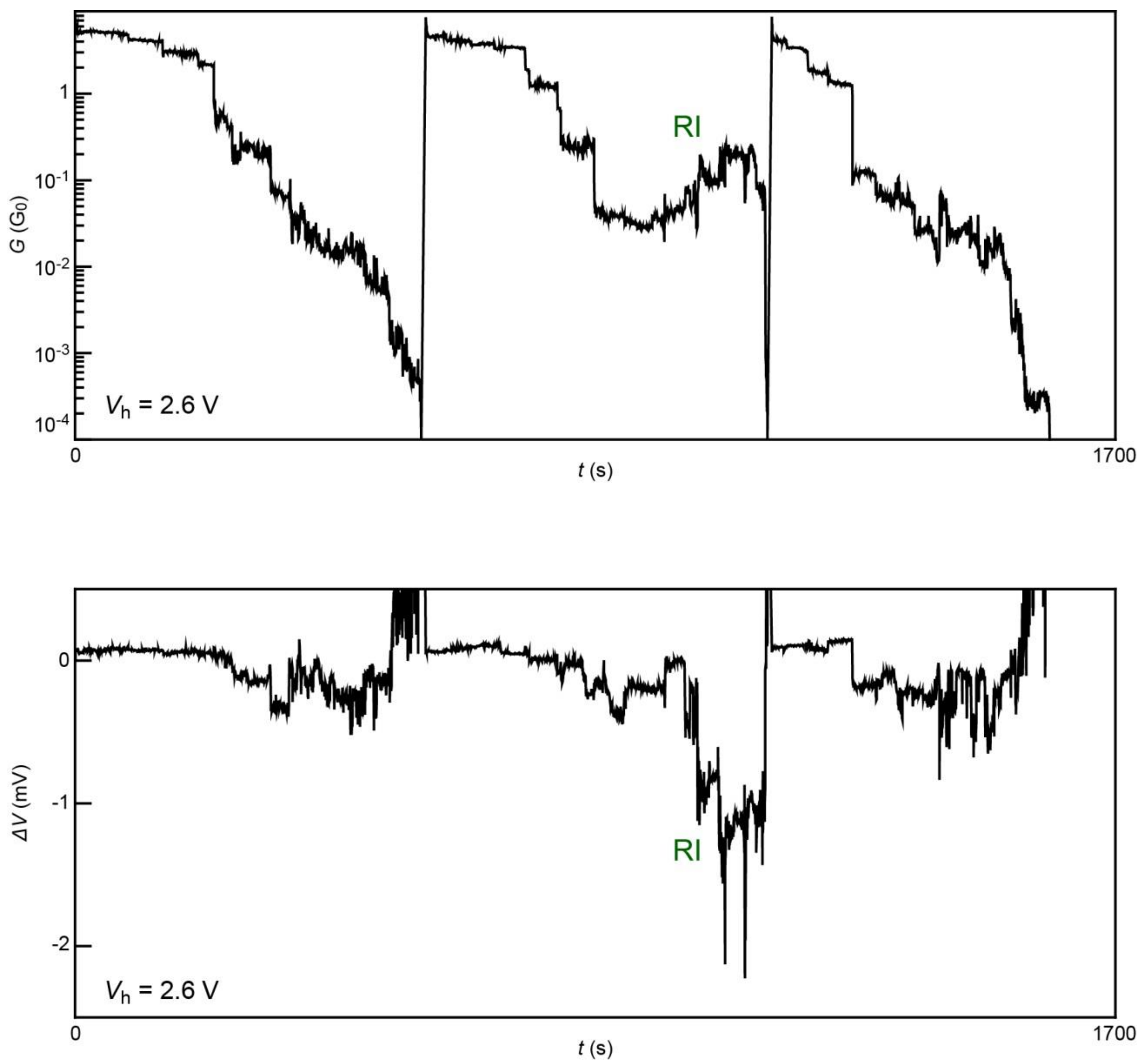

Figure S13. High-performance single-molecule thermoelectric junction. Three consecutive conductance (top) and thermoelectric voltage (bottom) traces at V_h = 2.6 V showing concomitant increase in G and ΔV during stretching (second curve).

5. Supplementary references

S1. van Ruitenbeek, J. M. et al. Adjustable nanofabricated atomic size contacts. *Rev. Sci. Instrum.* **67**, 108-111 (1996).

S2. Agraït, N.; Yeyati, A. L. & van Ruitenbeek, J. M. Quantum properties of atomic-sized conductors. *Phys. Rep.* **377**, 81-279 (2003).

S3. Vrouwe, S. A. G., van der Giessen, E.; van der Molen, S. J.; Dulic, D.; Trouwborst, M. L. & van Wees, B. J. Mechanics of lithographically defined break junctions. *Phys. Rev. B* **71**, 035313 (2005).

S4. Tsutsui, M.; Taniguchi, M. & Kawai, T. Local heating in metal-molecule-metal junctions. *Nano Lett.* **8** 3293-3297 (2008).

S5. Lörtscher, E.; Weber, H. B. & Riel, H. Statistical approach to investigating transport through single molecules. *Phys. Rev. Lett.* **98** 176807 (2007).

S6. Tsutsui, M.; Shoji, K.; Taniguchi, M. & Kawai, T. Formation and self-breaking mechanism of stable atom-sized junctions. *Nano Lett.* **8** 345-349 (2008).

S7. Tsutsui, M.; Kawai, T. & Taniguchi, M. Thermoelectricity in atom-sized junctions at room temperatures. *Sci. Rep.* **3**, 3326 (2013).

S8. Xiao, X.; Xu, B. & Tao, N. J. Measurement of single molecule conductance: Benzenedithiol and benzenedimethanethiol. *Nano Lett.* **4** 267-271 (2004).

S9. Xiang, D.; Jeong, H.; Kim, D.; Lee, T.; Cheng, Y.; Wang, Q. & Mayer, D. Three-terminal single-molecule junctions formed by mechanically controllable break junctions with side gating. *Nano Lett.* **13** 2809-2813 (2013).
